# β-Amyloid_1-42_, HIV-1_Ba-L_ (Clade B) Infection and Drugs of Abuse Induced Degeneration in Human Neuronal Cells and Protective Effects of Ashwagandha (Withania somnifera) and Its Constituent Withanolide A

**DOI:** 10.1371/journal.pone.0112818

**Published:** 2014-11-21

**Authors:** Kesava Rao Venkata Kurapati, Thangavel Samikkannu, Venkata Subba Rao Atluri, Elena Kaftanovskaya, Adriana Yndart, Madhavan P. N. Nair

**Affiliations:** 1 Department of Immunology, Institute of NeuroImmune Pharmacology, Herbert Wertheim College of Medicine, Florida International University, Modesto A. Maidique Campus, Miami, Florida, 33199, United States of America; 2 Human and Molecular Genetics, Herbert Wertheim College of Medicine, Florida International University, Modesto A. Maidique Campus, Miami, Florida, 33199, United States of America; Torrey Pines Institute for Molecular Studies, United States of America

## Abstract

Alzheimer's disease (AD) is characterized by progressive dysfunction of memory and higher cognitive functions with abnormal accumulation of extracellular amyloid plaques and intracellular neurofibrillary tangles throughout cortical and limbic brain regions. Withania somnifera (WS) also known as ‘ashwagandha’ (ASH) is used widely in Ayurvedic medicine as a nerve tonic and memory enhancer. However, there is paucity of data on potential neuroprotective effects of ASH against β-Amyloid (1–42) (Aβ) induced neuropathogenesis. In the present study, we have tested the neuroprotective effects of Methanol: Chloroform (3:1) extract of ASH and its constituent Withanolide A (WA) against Aβ induced toxicity, HIV-1_Ba-L_ (clade B) infection and the effects of drugs of abuse using a human neuronal SK-N-MC cell line. Aβ when tested individually, induced cytotoxic effects in SK-N-MC cells as shown by increased trypan blue stained cells. However, when ASH was added to Aβ treated cells the toxic effects were neutralized. This observation was supported by cellular localization of Aβ, MTT formazan exocytosis, and the levels of acetylcholinesterase activity, confirming the chemopreventive or protective effects of ASH against Aβ induced toxicity. Further, the levels of MAP2 were significantly increased in cells infected with HIV-1_Ba-L_ (clade B) as well as in cells treated with Cocaine (COC) and Methamphetamine (METH) compared with control cells. In ASH treated cells the MAP2 levels were significantly less compared to controls. Similar results were observed in combination experiments. Also, WA, a purified constituent of ASH, showed same pattern using MTT assay as a parameter. These results suggests that neuroprotective properties of ASH observed in the present study may provide some explanation for the ethnopharmacological uses of ASH in traditional medicine for cognitive and other HIV associated neurodegenerative disorders and further ASH could be a potential novel drug to reduce the brain amyloid burden and/or improve the HIV-1 associated neurocognitive impairments

## Introduction

Alzheimer's disease (AD) is the most prevalent neurodegenerative disease affecting approximately 36 million people worldwide [1] and if the current trend continues without medical innovation, one in 85 people will be affected with AD by 2050 [2]. Considerable attention has been focused on the deposition of insoluble β-amyloid peptide (Aβ) within the brain as a major etiologic factor in the pathogenesis of AD which is characterized by a decline in cognitive functions, for example memory loss, language deficit associated with behavioral and psychological symptoms like depression, stress, anxiety and mental upset [3], [4]. Pathological hallmarks include toxic β-amyloid plaques, neurofibrillary tangles, dystrophic neuritis, gliosis, decline of neurochemicals which are essential for neuronal transmission and neuroinflammation [5]–[7]. The Aβ cytotoxicity to neuronal cells has been identified as one of the major features in AD pathology, but the exact mechanisms involved leading to neurotoxicity still remain an enigma [8], [9]. A widely recognized concept about AD pathogenesis is the “amyloid hypothesis,” whereby augmented production and self-assembly of Aβ toxic constituents begins a sequence of advancing alterations that eventually lead to neuronal degeneration [10]–[13]. In this hypothesis, continuous Aβ toxicity associated stress activates the hyper-phosphorylation and aggregation of the microtubule-associated protein tau, resulting in neurofibrillary tangles, which are a major pathological hallmark of AD [12]. Accordingly, a better understanding of the mechanisms that are associated with the generation, accumulation and clearance of Aβ might represent a promising therapeutic approach for the treatment of AD. Neuronal degeneration is also a major feature in HIV infection and AIDS. Specifically, increased amyloid-β precursor protein (AβPP) in axons in the subcortical white matter tracts have been described by several investigators [14]–[16]. It has been reported that HIV persists in the brain during HAART therapy and that the local inflammatory responses to HIV in the brain could lead to increased AβPP production and susceptibility to Aβ deposition [17]. All these observations indicate that Aβ accumulation may be a good indicator of early neuronal (axonal) degeneration not only during the development of AD but also during HIV induced neuronal degeneration.

Withania somnifera (WS) “also known as ‘ashwagandha’ (ASH) in Sanskrit” is a multipurpose medicinal plant which has been used in a remarkable number of pharmacological studies in recent years, as it has been shown to possess a wide spectrum of therapeutic properties such as nerve tonic, memory enhancer, antistress, immunomodulatory and antioxidant properties [18], [19]. Withanolide A and withanoside IV from roots help to promote neurite outgrowth in cultured neurons and in rodents injected with Aβ 25–35 [20]. Root extracts from this species have also been shown to significantly reduce the number of hippocampal degenerating cells in the brains of stressed rodents [21] and were neuro-protective in animal models of Parkinson's disease [22]. A recent study of oral administration of a semi-purified extract of the root of ASH consisting predominantly of withanolides and withanosides reversed behavioral deficits, plaque pathology, accumulation of Aβ and oligomers in the brains of middle-aged and old APP/PS1 Alzheimer's disease transgenic mice [23]. However, there is a paucity of data on the molecular mechanisms associated with the potential protective effects of ASH root, as used traditionally, against Aβ-induced cytotoxicity and associated neuronal degeneration. Accordingly, we hypothesized that it will be of great interest to use ASH to reverse the neuronal toxicity induced by Aβ which may serve as a potential therapeutic agent for use in AD and possibly in other HIV related disorders involving memory deficiency. We have reported that Aβ induced cytotoxic effects in SK-N-MC cells when tested individually, however, when ASH was added to Aβ treated cultures, the cytotoxic effects of Aβ were neutralized [24]. In the present research, we wish to report that ASH and its constituent, Withanolide A (WA) can, not only prevent Aβ induced cytotoxicity and cell death, cellular localization, MTT formazan exocytosis and inhibition of acetylcholinesterase activity but also protect against HIV infection and neurodegenerative effects of substance abuse, indicating the neuroprotective properties.

## Materials and Methods

### Cell Culture

The effects of Aβ and ASH were tested on the human neuronal cell line, SK-N-MC, obtained from American Type Culture Collection (ATCC) (Catalog # HTB-10; Manassas,VA). The cells were grown in T-75 flasks containing Eagle's minimum essential medium (MEM) (GIBCO) with fetal bovine serum to a final concentration of 10% and 1% antibiotic/antimycotic solution. The cells were maintained in a humidified, 95% air and 5% CO_2_ atmosphere incubator at 37°C.

### Fibrillar β-Amyloid_1–42_ (Aβ_1–42_) or “seed” preparation

Fibrillar **Aβ_1–42_** was prepared as described by Wogulis et al [25]. One mg of Aβ_1–42_ lyophilized powder (Catalog # A9810, Sigma-Aldrich) was dissolved in 200 µl of water in glass vial and aged for 3 days at 37°C and was diluted with tissue culture medium to the required concentration, before adding to neuronal cultures.

### Extracts of Withania somnifera roots

The dried roots of ASH were purchased from an authenticated source in Kerala, India (Arya Vaidya Sala, Kottakal). The ground powder (15 g) was suspended in 300 ml of solvents (Methanol: Chloroform) (3:1), refluxed for 3 hr. and the supernatant collected. The residue was again suspended in 300 ml of same solvent and refluxed for another 3 hr. and the supernatant collected. Both the supernatants were combined, filtered to remove insoluble material and concentrated to dryness using a rotary vacuum evaporator. The dried extract was solubilized in dimethylsulfoxide (DMSO), aliquoted, stored at −20°C and utilized for all experiments. Analysis and identification of this dried extract using positive ion LS-MS showed the presence of Withanolide A as the major constituent, identified against a standard [24]. Besides Withanolide A, presence of some minor components was also observed [24]. Further studies are required to identify these components and their biological significance.

### Analysis of cell death in Aβ treated cultures

Cell death assays using logarithmically-growing cells were performed using the previously described protocol [26] with slight modifications. In brief, SK-N-MC cells were grown onto 22 mm ×22 mm glass coverslips at a concentration of 0.1×10^6^ in 3 ml of culture medium in 6-well plates for 48 hours and after that changed to 1 ml of serum free medium. ASH was added at 0.16 µg/ml to plain ASH control and Aβ plus ASH cultures. For ASH additions, DMSO served as the vehicle to dilute the compound at a final concentration of 0.4% volume per volume and at this concentration has no effect on cell survival. Control cultures received only solvent in the place of test compound. Three hours after pre-incubation of cells with ASH, Aβ and Aβ plus ASH cultures received Aβ at a concentration of 5 µM. Different investigators have used different dose levels of Aβ depending on the cell type utilized. Michaelis et al [27] used 5 and 10 µM concentration on cortical cell cultures whereas Yankner et al [28] used 20 µM on hippocampal neurons. Kumar et al [29] utilized 0.007-2 µg/mL concentration on PC12 cell line and London et al [30] 0.2, 2.0 and 20 µM on peripheral blood monocytes (PBM). We have standardized the doses required for our studies by testing at different doses and selecting a dose of about 40% inhibition and used for all our experiments [24]. After 24 hours, 10 µl of 0.4% Trypan Blue (GIBCO) stain solution were added to the culture medium of cells. After 15 min cells were washed with PBS and glass coverslips with cells were mounted on slides in glycerol/distilled water (1∶1) plus 0.1% sodium azide (Sigma-Aldrich) and the cells were examined under a microscope using an objective 40× (400x magnification) and photographed to detect stained cells.

### Internalization of Aβ _1–42_ determined by Congo red staining

Same protocol described above was utilized for culture conditions, addition of ASH and Aβ and exposure time. After the culture period the cells were washed with PBS, fixed in 4% formalin for 15 min at room temperature. Again cells were washed with PBS and then stained with a fresh alkaline solution of 0.5% filtered Congo red (Sigma-Aldrich) at room temperature for 5 min. After several washes with deionized water, slides were mounted in glycerol/distilled water (1∶1) plus 0.1% sodium azide (Sigma-Aldrich) and then observed under a microscope using an objective 40× (400x magnification) and photographed to detect stained cells.

### MTT Formazan Exocytosis

Same protocol described above for analysis of cell death was utilized for culture conditions, addition of ASH and Aβ and exposure time. After the culture period 100 µl of MTT (100 mg MTT/20 ml PBS) were added for each well and incubated at 37°C for 2 hours. After that cells were washed with PBS and glass coverslips with cells were mounted on slides in glycerol/distilled water (1:1) plus 0.1% sodium azide (SIGMA-ALDRICH) and the cells were examined under a microscope using an objective 40× (400x magnification) and photographed to detect MTT formazan exocytosis. The needle-like crystals on the surface of the cells treated with only Aβ, which are easily visible under a light microscope, represent exocytosed MTT formazan [31], [32].

### Acetylcholinesterase Activity

Determination of the acetylcholinesterse activity was carried out as per the method of Vinutha et al [33] with some modifications. In brief, cells were harvested from exponential phase cultures by trypsinisation, counted and plated in 96-well flat-bottomed microtitre plates at a seeding density of 3×10^4^ in 200 µl of medium for 24 hours and after that changed to 200 µl of serum free medium. ASH was added at 0.16 µg/ml to plain ASH control and Aβ plus ASH cultures. For ASH additions, DMSO served as the vehicle to dilute the compound at a final concentration of 0.4% volume per volume and at this concentration has no effect on cell survival. Control cultures received only solvent in the place of test compound. Three hours after pre-incubation of cells with ASH, Aβ and Aβ plus ASH cultures received Aβ at a concentration of 5 µM. After 48 hours, the medium was removed and a pre-incubation volume of 250 µl of phosphate buffer (200 mM, pH 7.7) containing 80 µl of DTNB (3.96 mg of DTNB and 1.5 mg of sodium bicarbonate dissolved in 10 ml of phosphate buffer, pH 7.7) was added to the cultures and incubated at room temperature for 5 min. Following pre-incubation, 15 µl of substrate (10.85 mg of acetylthiocholine iodide dissolved in 5 ml of phosphate buffer, pH 7.7) was added and incubated at 37°C for one hour. The enzyme acetylcholinesterase of the cells releases thiocholine from acetylthiocholine which reacts with dithiobisnitrobenzoic acid to produce yellow color which was measured in a microwell plate reader at 412 nm. The optical density of the yellow color is directly proportional to the enzyme activity and utilized for calculation of percent values.

### Effects of ASH on HIV-1 infection and drugs of abuse and Western blot analysis

SK-N-MC (1×10^6^) cells were cultured in T-75 flasks in 10 ml complete medium for 48 hours and after that changed to 10 ml of fresh complete medium and polybrene (5 µg/ml) was added for 8 hours. After that cells were infected with 100 ng of HIV-1_Ba-L_ (clade B) (NIH AIDS Reagent Program, Cat. # 510) overnight in respective flasks as described earlier [24]. Next day morning, cells were washed with PBS to remove unattached virus and replaced with fresh 10 ml medium and/or treated with drugs of abuse and ASH. The groups included are 1) Plain Controls, 2) HIV-1 Infected, 3) Cocaine Treated (100 nM), 4) Methamphetamine (METH) Treated (25 µM), 5) ASH was added at 0.16 µg/ml to plain ASH controls, 6) HIV-1+ ASH, 7) Cocaine + ASH, 8) METH + ASH, 9) HIV-1 + Cocaine + ASH and 10) HIV-1 + METH + ASH. For ASH additions, DMSO served as the vehicle to dilute the compound at a final concentration of 0.4% volume per volume and at this concentration has no effect on cell survival. Control cultures received only solvent in the place of test compound. For combination of HIV-1, ASH and drug effects, same concentrations in combination were utilized. The cells were cultured with additions for 48 hours and after that cells were washed with PBS solution and were harvested using Trypsin/EDTA solution (ScienceCell Laboratories), cell pellets were collected and lysed using lysis buffer (Pierce, IL) with 1x complete cocktail of protease inhibitors. Total cellular protein in equal quantity was resolved by 4–15% polyacrylamide gel electrophoresis, transferred to a nitrocellulose membrane. The following primary antibodies were used: anti-MAP2 (Cell Signaling Technology, Cat # 4542) and anti-GAPDH (Santa Cruz Biotechnology). Immunoreactive bands were visualized using a chemiluminescence's Western blotting system according to the manufacturer's instructions (Amersham). The supernatant obtained from the used medium was used for the p24 antigen estimation using ELISA kit (ZeptoMetrix Corp. Cat # 0801200).

### MTT Cell Viability Assay

The MTT cell viability assay was carried out by the modified assay as described by Kurapati et al. [24]. After the culture period, media was removed from the 6-well plates and one ml of PBS was added. A solution of 100 µl of MTT (100 mg MTT/20 ml PBS) was added for each well and incubated at 37°C for 3 hours. After that, one volume of stop mix solution (20% SDS in 50% *N, N*-Dimethylformamide) was added and rocked for about 2 hours and the optical density of the solubilized formazan was determined spectrophotometrically measuring the absorbance at 550 nm. The optical density of formazan in each well is directly proportional to the cell viability and utilized for calculations.

### Dose response curve and Neuroprotective effects of Withanolide A (WA)

The SK-N-MC (3×10^3^) cells obtained from sub-confluent flasks were cultured in 6-well plates in 2 ml complete medium for 48 hours and after that media were removed and equal volume of fresh medium was added. WA (Cat # W2145, Sigma-Aldrich) was added at different concentrations in 8 µl volumes and cultured for another 48 hours. For WA additions, DMSO served as the vehicle to dilute the compound at a final concentration of 0.4% volume per volume and at this concentration has no effect on cell survival. Control cultures received only solvent in the place of test compound. After the culture period, cells were utilized for MTT assay as described above. The neuroprotective effects of WA were performed using the previously described protocol [24]. SK-N-MC cells were grown at a concentration of 3×10^3^ in 2 ml of culture medium in 6-well plates for 48 hours and after that changed to 1 ml of serum free medium. WA was added at two doses of 10 µg/ml and 20 µg/ml to plain WA control and β-amyloid plus WA cultures. Control cultures received only solvent in the place of test compound. Three hours after pre-incubation of cells with WA, Aβ and Aβ plus WA cultures received Aβ at a concentration of 5 µM [24]. After 24 hours, cells were utilized for MTT assay as described above.

### Effects of WA on HIV-1 infection and drugs of abuse and MTT assay

SK-N-MC (3×10^3^) cells were cultured in 6-well plates in 2 ml complete medium for 48 hours and after that changed to 2 ml of fresh complete medium and polybrene (5 µg/ml) was added for 8 hours. After that, cells were infected with 25 ng of HIV-1_Ba-L_ (clade B) (NIH AIDS Reagent Program, Cat. # 510) overnight as described earlier [24]. Next day morning, cells were washed with PBS to remove unattached virus and replaced with fresh 2 ml medium and/or treated with drugs of abuse and WA. The groups included are 1) Plain Controls, 2) HIV-1 Infected, 3) Cocaine Treated (100 nM), 4) Methamphetamine (METH) Treated (25 µM), 5) WA was added at 20.0 µg/ml to plain WA controls, 6) HIV-1+ WA, 7) Cocaine + WA, 8) METH + WA, 9) HIV-1 + Cocaine + WA and 10) HIV-1 + METH + WA. For WA additions, DMSO served as the vehicle to dilute the compound at a final concentration of 0.4% volume per volume and at this concentration has no effect on cell survival. Control cultures received only solvent in the place of test compound. For combination of WA, HIV-1 and drug effects, same concentrations in combination were utilized. The cells were cultured with additions for 48 hours and after that media removed and cells were washed with PBS solution and were utilized for MTT assay as described above. The supernatant obtained from the used medium was used for the p24 antigen estimation using ELISA kit (ZeptoMetrix Corp. Cat # 0801200).

### Statistical Analysis

The results are expressed as mean ± standard deviation and the significance was evaluated using the Student's t-test (GraphPad Software, CA, USA). Results giving p≤0.02 were considered significant.

## Results

Chemoprevention has been acknowledged as an important and practical strategy for the management of several disorders. From this point of view, we have selected root extract of ASH, which is in common use in Indian traditional medicine to promote cognition, including memory. The major chemical constituents of the roots are withanolides, particularly WA, although the mechanism by which WS root accomplishes these activities is still largely unknown. Accordingly, the root powder was extracted with a mixture of methanol: chloroform (3:1) for testing the beneficial effects on SK-N-MC, a neuronal cell line. We have also tested its purified constituent WA. This extract [24] and its purified product WA showed growth stimulatory effects on SK-N-MC cell line and accordingly was used for all our studies.

### Analysis of cell death in Aβ treated cultures

**Figure 1:**
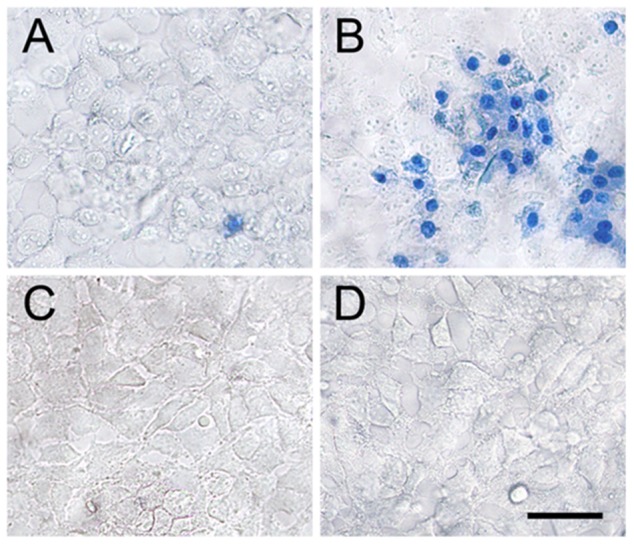
Analysis of β-Amyloid toxicity and its reversal by Ashwagandha. Untreated, ashwagandha and ashwagandha plus β-Amyloid treated cells exhibited good viability as evidenced by exclusion of trypan blue dye, contrary to the positive trypan blue staining of SK-N-MC cells treated with β-Amyloid. A. Control, B. β- Amyloid _1-42_ treated, C. Ashwagandha treated and D. Ashwagandha plus β- Amyloid_1-42_ treated. Scale bar: 100 µm. Experiment was repeated three times.

The trypan blue staining of untreated SN-N-MC cells showed mostly viable cells with very few trypan-positive cells (Fig.1A). In contrast, the plain Aβ treated cultures stained significant number of trypan-positive cells (Fig.1B) suggesting that Aβ treatment promoted cell degeneration and death compared to plain controls. However, when ASH was added to Aβ treated cultures, the cytotoxic effects of Aβ were neutralized (Fig.1D) and the cells were comparable to plain (Fig.1A) and ASH (Fig.1C) treated controls, indicating the chemopreventive or protective effects of ASH against Aβ induced toxicity.

### ASH decreases the internalization of Aβ _1–42_

**Figure 2:**
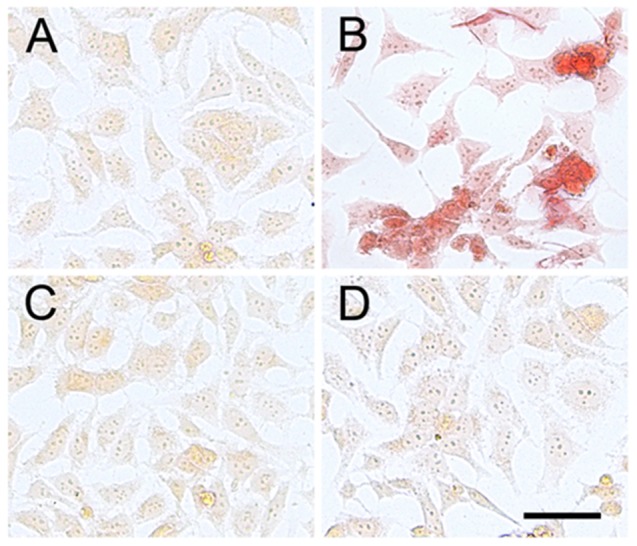
Congo red staining showing the increased internalization in β- Amyloid_1-42_ treated and its reversal by Ashwagandha in SK-N-MC cell line. A. Control, B. β- Amyloid _1-42_ treated, C. Ashwagandha treated and D. Ashwagandha plus β- Amyloid_1-42_ treated. Scale bar: 100 µm. Experiment was repeated three times.

In order to understand the effect of ASH on the internalization of Aβ in SK-N-MC cells, cells were pre-incubated with the extract for three hours and then exposed to Aβ for 24 hours. After that, cells were stained with Congo red, a metachromatic anionic dye, specific for Aβ. As Fig.2 shows, cultures treated with Aβ alone (Fig.2B) showed much more marked internalization of the toxic peptide than occurred when cells were incubated with Aβ plus ASH (Fig.2D). Plain controls (Fig.2A) and ASH (Fig.2C) treated cultures showed only back ground staining.

### MTT Formazan Exocytosis by β-Amyloid Treatment

**Figure 3:**
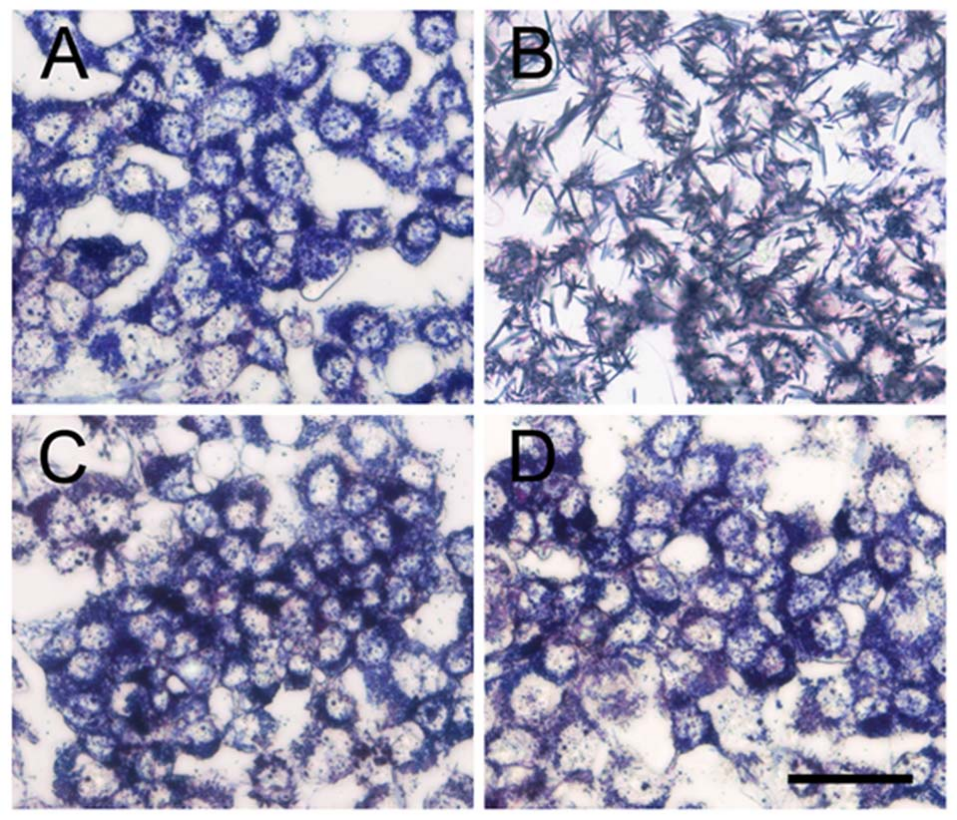
Effect of β-Amyloid toxicity on the appearance of needle-like MTT formazan exocytosis and its reversal by Ashwagandha. Untreated, ashwagandha and ashwagandha plus β-Amyloid treated cells showed even intracellular distribution of formazan crystals, contrary to the MTT formazan exocytosis and needle-like formazan crystals on the cell surface of SK-N-MC cells treated with β-Amyloid. A. Control, B. β- Amyloid _1-42_ treated, C. Ashwagandha treated and D. Ashwagandha plus β- Amyloid_1-42_ treated. Scale bar: 100 µm. Experiment was repeated three times.

When viable cells are incubated with the tetrazolium salt MTT, they reduce the salt to a purple water-insoluble formazan. Reduction of MTT in cells is regarded as an indicator of “cell redox activity” and the reaction is attributed mainly to mitochondrial enzymes besides number of non-mitochondrial enzymes present in the endoplasmic reticulum, cytosol and plasma membranes. When examined under a microscope, formazan granules of reduced MTT are confined to different intracellular organelles. However, in cells treated with Aβ, needle-like formazan crystals begin to appear on the cell surface, which are the exocytosed MTT formazan [32] and clearly visible under the light microscope. The effect of MTT cellular reducing activity on the images of morphological characteristics and architecture in Aβ (Fig. 3B) as well as Aβ plus ASH (Fig.3D) and control (Fig.3A) and only ASH (Fig.3C) treated SK-N-MC cells were captured using light microscopy. Treatment of SK-N-MC cells with Aβ resulted in significant increase in MTT formazan exocytosis and needle-like formazan crystals on the cell surface (Fig.3B) compared with the even intracellular distribution in untreated control cells (Fig.3A). Also, both ASH (Fig.3C) and ASH plus Aβ (Fig.3D) treated cells showed no formazan exocytosis and were comparable with untreated control (Fig.3B).

### Increased Acetylcholinesterase Activity in β-amyloid treated cultures and its reversal by ashwagandha

**Figure 4:**
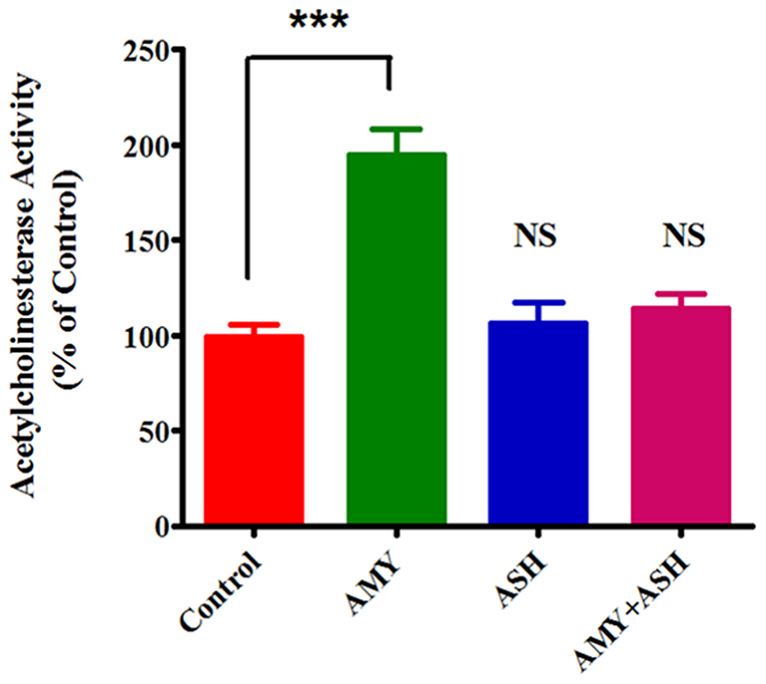
Increased acetylcholinesterase activity by β-Amyloid and its reversal by ashwagandha. The values were expressed as percentage of acetylcholinesterase activity in control cells and are the mean ± SD of five experiments. *** indicates a statistically significant difference compared to controls (p<0.0001). ASH - Ashwagandha; AMY - β-amyloid.

It is known that one of the characteristic alterations that occur in Alzheimer's disease is the loss of acetylcholinesterase (AChE) activity, the enzyme that is responsible for acetylcholine hydrolysis, from both cholinergic and non-cholinergic neurons of the brain. However, AChE activity has been shown to be increased within and around amyloid plaques to promote the assembly of amyloid beta-peptides into fibrils and to increase the cytotoxicity of these peptides suggesting that AChE could play a pathogenic role in AD by influencing the process leading to amyloid toxicity. Therefore, in order to understand the role of AChE during the exposure of Aβ as well as in the combination of Aβ plus ASH, acetylcholinesterase activity was determined in cells after exposure. The activity of AChE, the enzyme responsible for the acetylcholine degradation was significantly increased (p<0.0001) in cells treated with Aβ (0.267±0.011) compared with the untreated control cells (0.17±0.15) (Fig.4). In ASH controls and as well as ASH plus Aβ treated cells, the AChE activity was not different compared to untreated control (Fig.4).

### Increased MAP2 protein levels in HIV-1 infected and drugs of abuse treated cultures and their reversal by ASH

**Figure 5:**
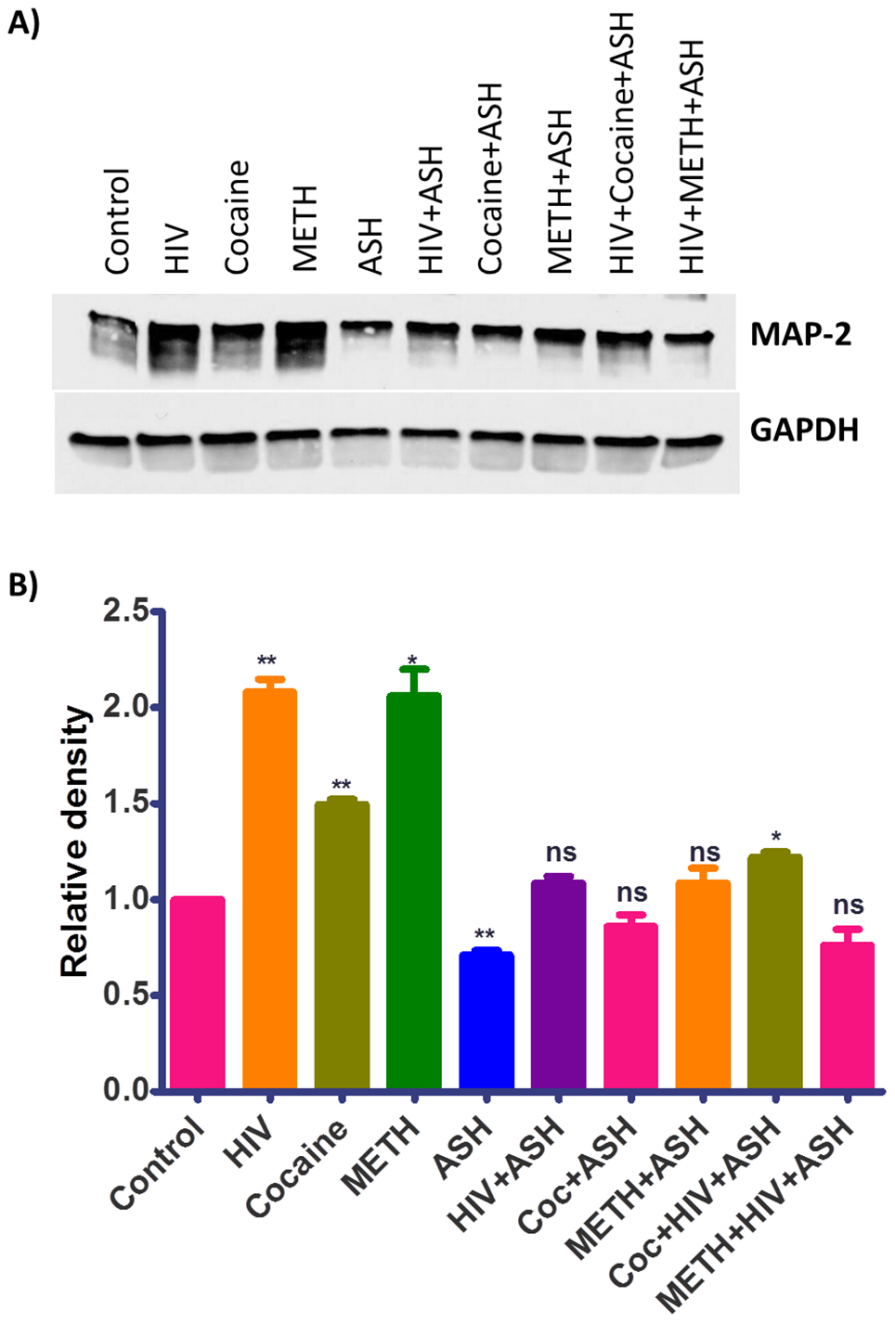
Western blotting analysis showing the increased MAP2 protein levels in HIV-1_Ba-L_ (clade B), Cocaine and METH treated and their reversal by Ashwagandha in SK-N-MC neuronal cells. (A) Cell lysates were separated in 4% to 15% linear gradient SDS-PAGE gels and were probed against the respective antibodies. GAPDH was used as the loading control. (B) Quantitative analysis showing the increased MAP2 protein levels in HIV-1_Ba-L_ (clade B), Cocaine and METH treated and their reversal by Ashwagandha. The gel shown is a representative for three experiments.

Microtubule-associated protein 2 (MAP2) is a marker of neuronal differentiation that regulates the structure and stability of microtubules. Dynamic instability of microtubules is critical for mitotic spindle assembly and disassembly during cell division, especially in rapidly dividing cells. Microtubule-associated proteins (MAPs) are a family of proteins that influence this property. MAP2 stabilizes microtubules and that disrupts the dynamic instability of microtubules which inhibit cell division. It has been reported that in metastatic melanoma cell lines MAP2 expression induces microtubule stabilization, cell cycle arrest in G2-M phase and growth inhibition. Further, disruption of microtubule dynamics by MAP2 resulted in multipolar mitotic spindles, defects in cytokinesis and accumulation of cells with large nuclei [34]. This suggests the possible pathogenic role of MAP2 in neurodegeneration due to its increased levels. Accordingly, in order to understand the neurodegenerative role of MAP2 during HIV-1 infection and as well as during the exposure to drugs of abuse and their possible reversal by ASH, MAP2 levels were determined in cells after their exposure by Western blotting. The levels of MAP2 were significantly increased in cells infected with HIV-1and as well as in cells treated with Cocaine and METH compared with the untreated control cells (Fig.5). In plain ASH treated cells the MAP2 levels were significantly less compared to controls (Fig.5). In cells treated with combination of HIV-1+ ASH, Cocaine + ASH, METH + ASH, and as well as HIV-1 + Cocaine + ASH and HIV-1 + METH + ASH, the MAP2 levels were reduced and comparable with controls (Fig.5).

### Dose response curve and Neuroprotective effects of Withanolide A (WA)

**Figure 6:**
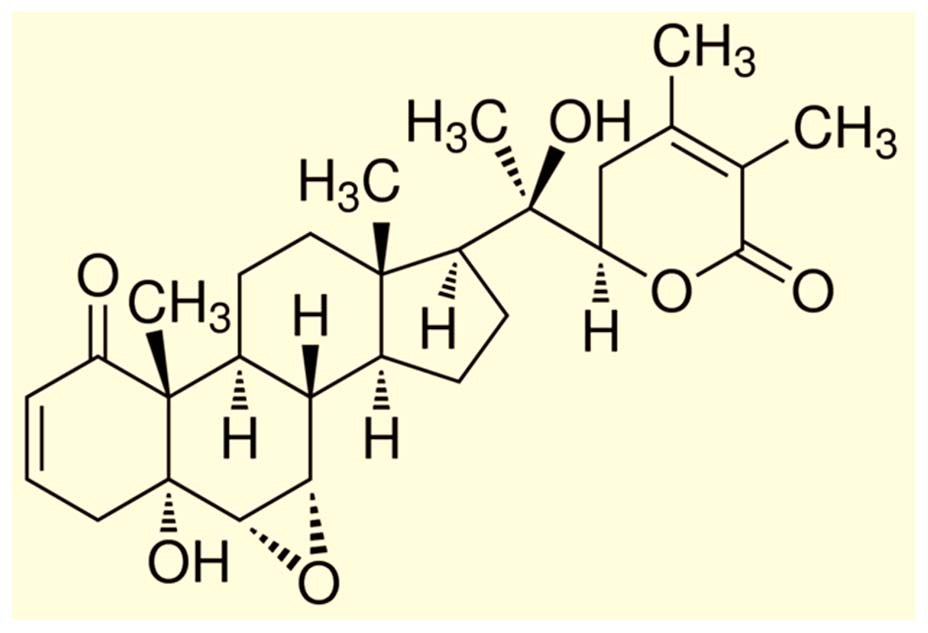
Chemical structure of Withanolide A.

**Figure 7:**
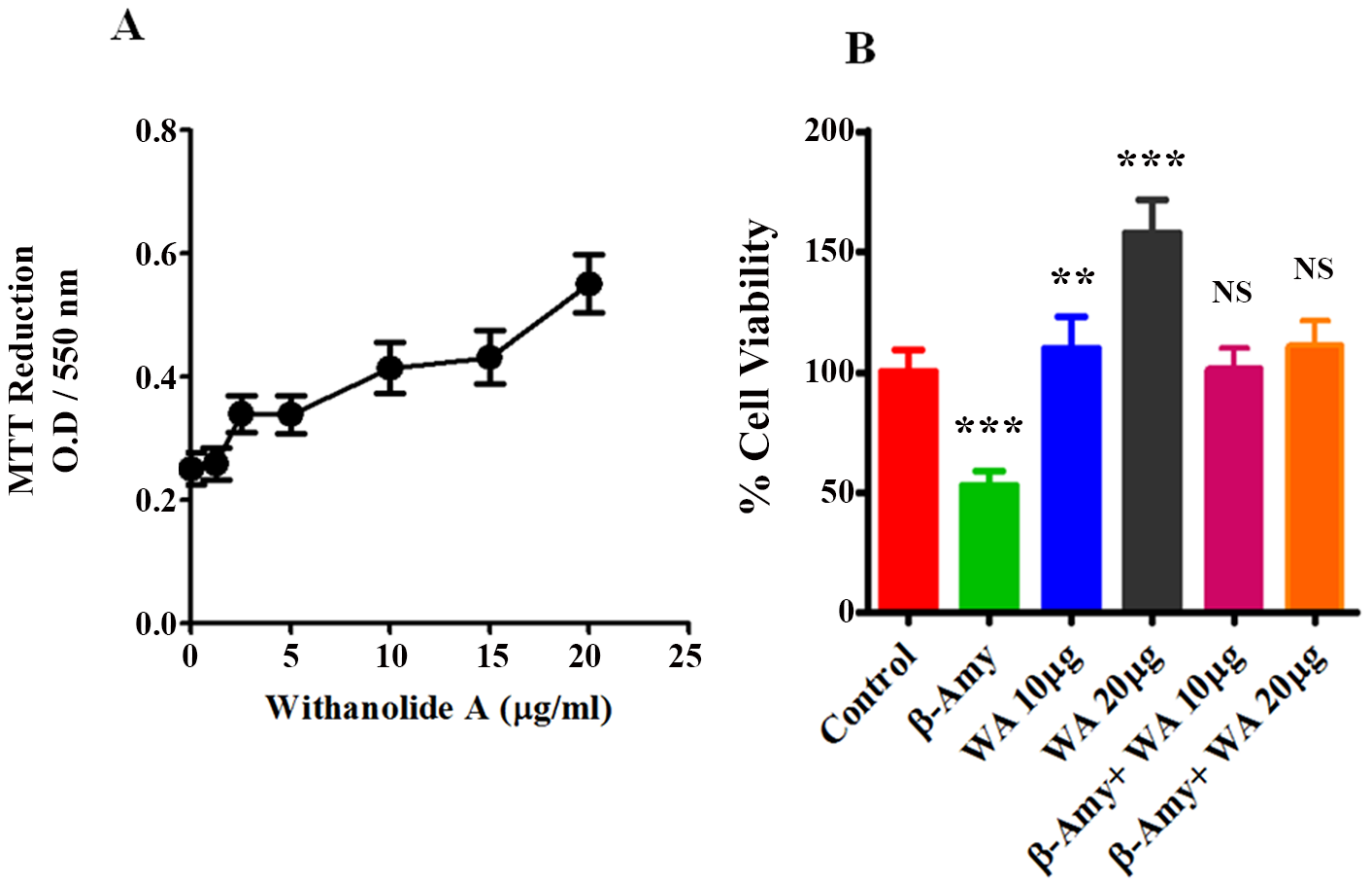
Modulatory effects of Withanolide A and β-Amyloid_1-42_ on human neuronal cells. A. Dose-response of Withanolide A showing growth-stimulatory effects in terms of % viability. B. MTT assay showing the inhibition of cell viability by β-Amyloid_1-42_ and its reversal by Withanolide A at two concentrations on SK-N-MC cells. The values were expressed as percentage of cell viability compared to control cells and are the mean ± SD of four experiments. *** and ** indicates a statistically significant difference (p<0.0001) and (p<0.0018) respectively compared to controls. WA – Withanolide A; β-AMY - β-Amyloid_1-42_

There is a significant need for the development novel drugs using small molecules that enhance and improve the memory and learning by influencing the cellular pathways which have lasting disease modifying effects that could alleviate the course of neurodegenerative diseases such as AD. We have reported recently, that Methanol: Chloroform (3:1) extract prepared from the dried roots of ASH, when subjected to LC-MS analysis showed the presence of a major peak of Withanolide A (WA), and the extract was neuroprotective against Aβ induced cytotoxicity and HIV-1 infection [24]. Since the major constituent ASH is WA, we wanted to see whether purified WA (Cat # W2145, Sigma-Aldrich, Mol.Wt. 470.60, Purity ≧95%), a C5, C6-epoxy steroidal lactone (Fig.6), exerts similar neuroprotective effects. WA has been shown earlier to possess strong neuropharmacological activities in promoting neurite outgrowth, reversing neuritic atrophy, and aiding synapse reconstruction [20], [37], [38] and thus provides a strong pharmacological rationale for further investigation. Figure 7A shows the dose-response curve for WA on SK-N-MC cells. WA exhibited a significant (p<0.0001) dose-dependent increase in cell viability as reflected by MTT activity from 1.25 µg/ml (0.267±0.035) to 20 µg/ml (0.573±0.5) compared to control (0.258±0.035). Figure 7B shows the histograms of cell viability in control, Aβ, WA and Aβ plus WA treated cultures at two different WA concentrations. The results supported the earlier observations that Aβ exerts cytotoxic effects in neuronal cells with decreased cell viability when tested individually. However, when WA was added to Aβ treated cultures, the cytotoxic effects of Aβ were neutralized thus showing the beneficial effects (Fig.7B).

### Effects of WA on HIV-1 infection and drugs of abuse and MTT assay

**Figure 8:**
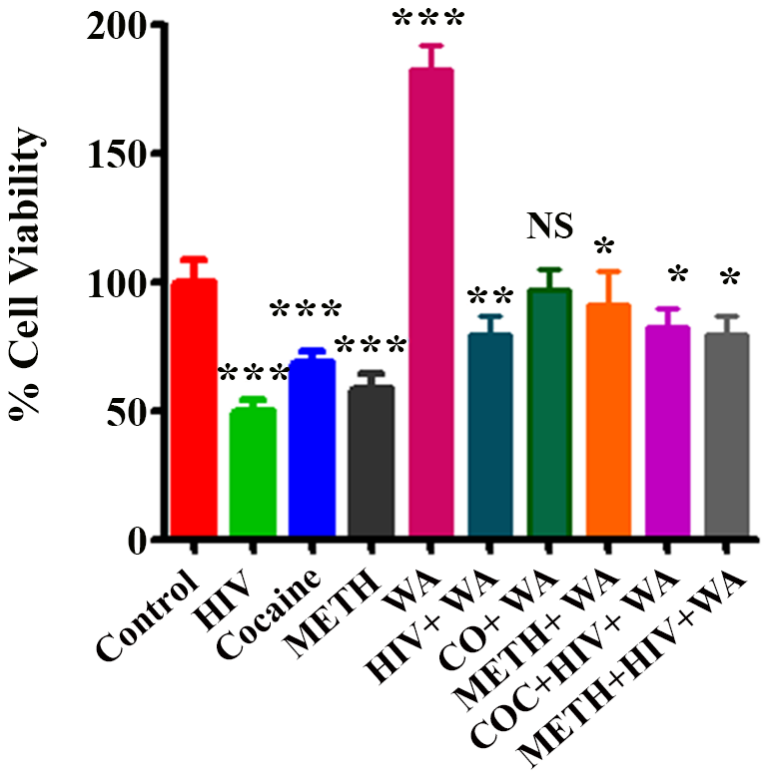
Protective effects of Withanolide A from HIV-1_Ba-L_ (clade B) infection, Cocaine and METH treated in SK-N-MC cells. MTT assay showing the inhibition of % cell viability in HIV-1_Ba-L_ (clade B), Cocaine and METH treated and their reversal by Withanolide A. Withanolide A alone showed significant increase in cell viability. The values were expressed as percentage of cell viability compared to control cells and are the mean ± SD of four experiments. ***, **, * indicates a statistically significant difference (p<0.0001), (p<0.0006), and (p<0.0185), respectively compared to controls. WA–Withanolide A; CO, COC – Cocaine; METH – Methamphetamine.

Neurodegenerative diseases commonly induce irreparable damage of central nervous system (CNS) neuronal networks, resulting in permanent functional impairments. Effective medications against neurodegenerative diseases are currently lacking. Neuroprotective properties of ASH are attributed to withanolides, particularly WA and therefore WA forms important candidate for the therapeutic treatment of neurodegenerative diseases like Alzheimer's disease. Further, WA is one of the most commonly found and extensively studied active ingredients of ASH and has attracted interest due to its strong neuropharmacological properties of promoting outgrowth and synaptic reconstruction [20], [37], [38]. Accordingly, the effects of WA were tested on SK-N-MC cells in order to understand the beneficial effects and see whether WA could protect cells from HIV-1 infection as well as during the exposure to drugs of abuse. Figure 8 shows the histograms of % cell viability in control, HIV-1, Cocaine, Methamphetamine, WA, in cells treated individually and in combination. The levels of % cell viability were significantly decreased (p<0.0001) in cells infected with HIV-1and as well as in cells treated with Cocaine and METH compared with the untreated control cells (Fig.8). In plain WA treated cells the % cell viability level was significantly elevated (p<0.0001) compared to control (Fig.8). In cells treated with combination of HIV-1+ WA, Cocaine + WA, Meth + WA, as well as HIV-1 + Cocaine + WA and HIV-1 + Meth + WA the % cell viability levels were relatively increased and comparable with controls (Fig.8).

## Discussion

Considerable attention has been focused on the accumulation of β-amyloid peptide (Aβ) within the brain as a major etiologic factor in the pathogenesis of Alzheimer disease (AD). At present, there is no curative treatment available for AD and the approved medications are used only for slowing the disease progression or providing prophylaxis. Numerous studies over the past two decades indicated that ASH has anti-inflammatory, anti-tumor, anti-stress, anti-oxidant, mind boosting and rejuvenating properties [35], [36]. Studies also showed that extracts of ASH promote dendrite formation in human neuroblastoma cells in vitro in a dose-dependent manner [37]. In our earlier studies, ASH extract showed growth stimulatory effects at relatively lower concentrations on SK-N-MC, a neuronal cell line [24]. Accordingly, this extract was utilized for identification of the components present as well as for other studies. HPLC and mass spectra analysis showed the presence of withanolide A as the main withanolide in the extract [24]. Withanolides, more particularly WA are known to be responsible for the multiple medicinal properties of ASH [38].

Many studies have utilized in vitro models to evaluate the toxicity and neurodegeneration in cells treated with Aβ, still, there is lack of information regarding the mechanisms involved during the neurodegenerative processes. In the present study, the Aβ treated cells, when stained with trypan blue showed several positive cells indicating cell death and cytotoxic effects compared to plain controls (Fig.1A and Fig.1B). However, when ASH was added to Aβ treated cultures, the cytotoxic effects of Aβ were neutralized (Fig.1D) and the cells were comparable to plain (Fig.1A) and ASH (Fig.1C) treated controls, suggesting the chemopreventive or protective effects of ASH against Aβ induced toxicity. Toxicity mediated by excitatory amino acids (EAAs) is a well-documented mechanism of neurodegeneration that has also been postulated to function in Aβ-induced toxicity [39], [40]. It is possible that similar mechanisms may be responsible for the cell degeneration observed in the present study during the exposure of Aβ.

Accumulating evidence from different clinical studies, transgenic models as well as in vitro studies suggests that intra-neuronal accumulation of Aβ as an early event that plays an important role in the pathogenesis of AD [41]. Further, extracellular addition of Aβ to neuronal cells in culture is known to induce the uptake of Aβ and its localization to the nucleus [42], [43]. In our study, cultures treated with Aβ alone (Fig.2B) showed much more marked internalization of the toxic peptide compared to cultures treated with Aβ plus ASH (Fig.2D). The plain (Fig.2A) and ASH (Fig.2C) controls showed background stain. It is likely that enhanced accumulation of Aβ in neuronal cells might potentiate the peptide's toxic effects and were counteracted by ASH as observed earlier, confirming the neuro-protective effects of ASH.

In cell culture models, 3-(4, 5-dimethylthiazol-2-yl)-2, 5-diphenyltetrazolium bromide (MTT), is widely used to elucidate the cellular toxicity of the Aβ peptide. It is generally believed that inhibition of MTT reduction by Aβ is an early indication of the Aβ-induced impairment of the cellular reducing activity. Further, it has recently been reported that Aβ inhibits cellular MTT reduction by enhancing MTT formazan exocytosis rather than by inhibiting MTT reduction directly [31], [32]. In this study, on microscopic examination, SK-N-MC cells treated with Aβ and incubated with MTT showed marked appearance of needle-like exocytosed formazan crystals on the cell surface (Fig.3B) compared with the intracellular even formazan granules seen in the untreated control cells (Fig.3A). Also both the Aβ plus ASH (Fig.3D) as well as plain ASH (Fig.3C) treated cells showed intracellular even formazan granules indicating the protective and reversal effect of ASH on MTT exocytosis. These observations suggest that Aβ enhances MTT formazan exocytosis probably through an intracellular signal transduction pathway which is attenuated by ASH.

Increasing evidence suggests that Aβ has a direct influence on cholinergic activity. Acetylcholinesterase activity is increased in plaques and tangles very early in the process of amyloid deposition. In particular, Aβ has been shown to induce the expression of acetylcholinesterase in the brains of CT-100-expressing transgenic mice and in cell culture experiments indicating a link between Aβ production and acetylcholinesterase activity. In our study, the results demonstrated that exposure of SK-N-MC cells to Aβ resulted in a significant (p<0.0001) increase in acetylcholinesterase activity (Fig.4) indicating cytotoxicity compared to controls. However, ASH plus Aβ treated cultures showed protective effects against the cytotoxicity as the levels of acetylcholinesterase activity were comparable with plain and ASH treated controls (Fig.4). It has been reported that Aβ enhances acetylcholinesterase activity by increasing calcium entry through L-type voltage-dependent calcium channels [44], suggesting that increase may be a consequence of a disturbance of calcium homeostasis.

Brain deposition of Aβ is a common pathologic feature in HIV positive patients. An increased prevalence of amyloid plaques was observed in cortex of the brains of AIDS patients compared with the age matched controls [45]. Several reports have shown an increase in the amyloid deposition in brains of HIV-1 infected patients [46]–[48]. Further, HIV-1 associated neurocognitive disorders (HAND) in elderly is associated with β-amyloidosis and that the Aβ accumulation reflects the clinical outcome of HIV-1 infection [49]. Due to higher survival rates after antiretroviral therapy (ART), several factors including aging, HIV-1 infection, and secondary effects of ART contribute to brain Aβ deposition and further to AD. Illicit or drugs of abuse such as Methamphetamine (METH), and Cocaine are powerful psycho-stimulants that are widely abused in USA and all over the world and are significant risk factors for HIV-1 infection and AIDS disease progression [50]. Accordingly, both, HIV-1 infection and drugs of abuse contribute to the amyloid deposition in the brain and associated neurocognitive disorders and impacts in neurodegeneration. In the present study, the levels of MAP2 were significantly increased in cells infected with HIV-1and as well as in cells treated with Cocaine and METH compared with untreated control cells (Fig.5). In plain ASH treated cells the MAP2 levels were significantly less compared to controls. In cells treated with combination of HIV-1+ ASH, Cocaine + ASH, METH + ASH, and as well as HIV-1 + Cocaine + ASH and HIV-1 + METH + ASH, the MAP2 levels were reduced and comparable with controls (Fig.5). It is known that increased levels of MAP2 induces neuronal differentiation and can profoundly affect cell cycle progression and further induce apoptosis [34] and accordingly contribute to neurodegeneration. ASH and its constituents showed various beneficial effects against models of Alzheimer's disease and spinal cord injury. ASH extracts also showed ameliorative effects against other neurodegenerative disease models such as Parkinson's disease and Huntington's disease suggesting that ASH may be useful against various neurodegenerative diseases. The reduction in the levels of MAP2 in ASH treated cells and further decreasing the levels in HIV-1 infected and drugs of abuse treated cells suggests that ASH could be a potential novel drug to reduce the brain amyloid burden and/or improve the HIV-1 associated neurocognitive impairments.

ASH is recommended as “Medhya-Rasayana” (memory and intellect enhancers) in the ayurvedic traditional Indian medicinal system [51]. We have reported recently, that Methanol: Chloroform (3:1) extract prepared from the dried roots of ASH, when subjected to LC-MS analysis showed the presence of a major peak of Withanolide A (WA). Further, this ASH extract showed neuroprotective effects against β-amyloid induced cytotoxicity, HIV-1 infection, as well as drugs of abuse. Since the major constituent ASH is WA, we wanted to see whether purified WA (Cat # W2145, Sigma-Aldrich, Mol.Wt. 470.60, Purity ≧95%), a C5, C6-epoxy steroidal lactone (Fig.6), exerts similar neuroprotective effects. WA has been shown earlier to possess strong neuropharmacological activities in promoting neurite outgrowth, reversing neuritic atrophy, and aiding synapse reconstruction [20], [37], [38] and thus provides a strong pharmacological rationale for further investigation. Figure 8 shows the histograms of % cell viability in control, HIV-1, Cocaine, Methamphetamine, WA, in cells treated individually and in combination. The levels of % cell viability were significantly decreased (p<0.0001) in cells infected with HIV-1and as well as in cells treated with Cocaine and METH compared with the untreated control cells (Fig.8). In plain WA treated cells the % cell viability level was significantly elevated (p<0.0001) compared to the control. In cells treated with combination of HIV-1+ WA, Cocaine + WA, Meth + WA, as well as HIV-1 + Cocaine + WA and HIV-1 + Meth + WA the % cell viability levels were relatively increased and comparable with controls (Fig.8). These results suggest that WA has a potential basis for novel drugs against neurodegenerative diseases. However, direct target molecules of WA have not been identified clearly as yet. According to the “amyloid cascade hypothesis”, a chronic imbalance between the production and clearance of Aβ results in the formation of Aβ plaques and plays a major role in the etiopathogenesis of AD [52]. Aβ is generated via cleavage of the amyloid –β precursor protein (AβPP). The proteolytic processing of AβPP takes place by sequential cleavage by various proteases namely, α-, β-, and γ-secretase. It has been reported that β-secretase (BACE1) is up-regulated significantly in the AD brains and that WA significantly downregulated BACE1 levels in primary rat cortical neurons [53]. BACE1 is a rate-limiting enzyme in the production of Aβ and it has been reported that a slight increase in BACE1 levels leads to a dramatic increase in the production of Aβ 40/42 [54], [55]. Accordingly, it is possible that even a slight decrease in BACE1 levels may lead to a considerable decrease in the production of Aβ. Thus, WA with its substantial inhibitory activity against BACE1 may form a potentially effective novel compound for AD treatment by decreasing Aβ generation and deposition. Thus, modulation of one or more Aβ-degrading enzymes may prove vital in the prevention and treatment of AD.

**Figure 9:**
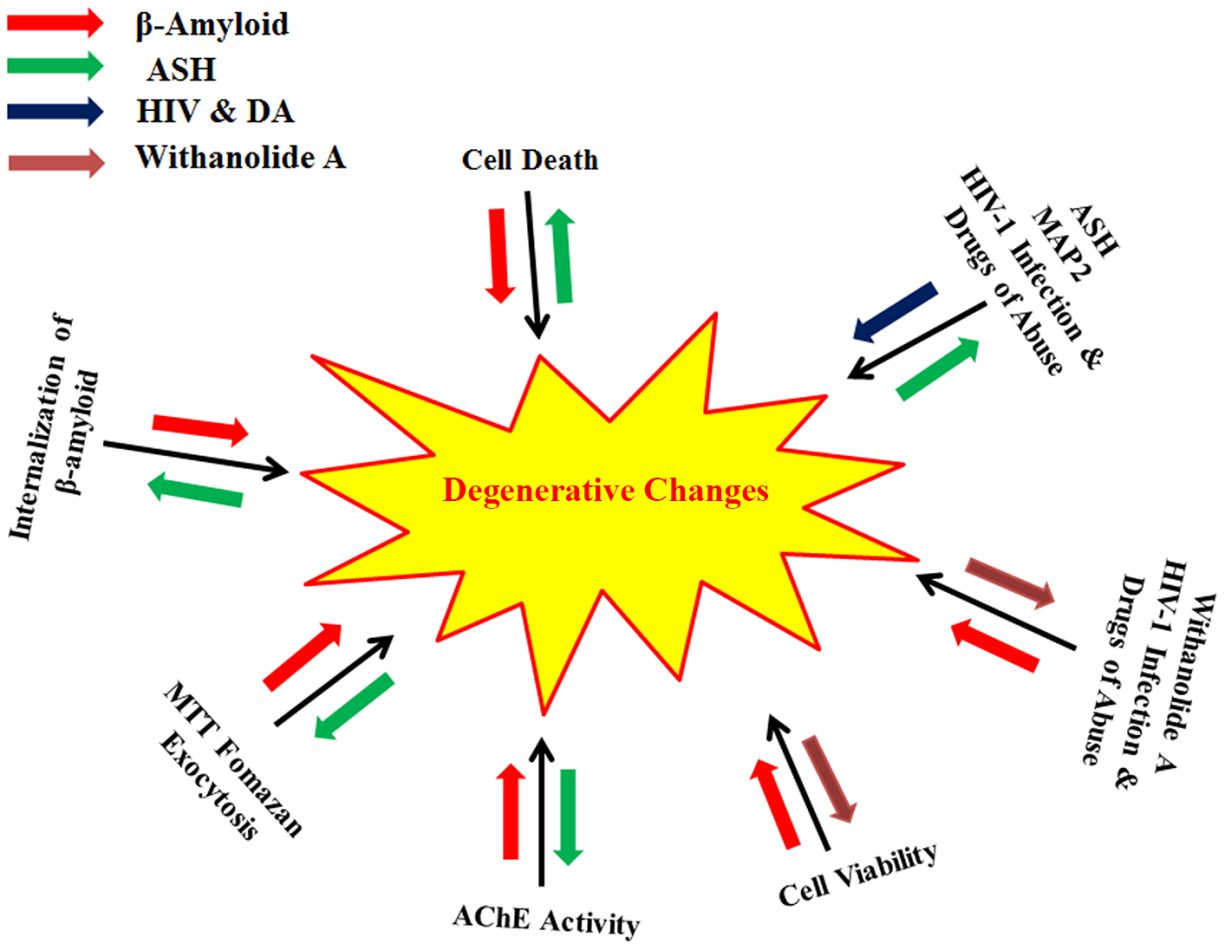
A comprehensive model showing the β-Amyloid, HIV-1_Ba-L_ (clade B) as well as drugs of abuse induced degenerative changes in SK-N-MC neuronal cells and their reversal by Ashwagandha (ASH) and Withanolide A (WA). Red and Blue arrows indicate increase in degenerative changes by β-Amyloid, HIV-1 and Drugs of Abuse respectively. Green and Orange arrows indicates reversal of degenerative changes by ASH and WA.

Even though, the mechanisms of AD are still not completely understood, it is believed that excessive accumulation of Aβ through abnormal β-amyloid precursor protein (AβPP) and Aβ metabolism are key events in the pathogenesis of AD. Thus, strategies targeting Aβ metabolism and AβPP processing are of immense help for the treatment and prevention of AD. Here we have demonstrated that ASH extract reverses the Aβ induced neuronal toxicity in SK-N-MC cells and may serve as a potential therapeutic agent for use in AD and possibly in other HIV related disorders involving memory deficiency (Fig.9). Some studies suggest that one pathway of Aβ induced cytotoxicity could be mediated by free radicals and oxidative stress [29]. It is known that ASH has antioxidant and free radical scavenger activity and this could inhibit Aβ induced cellular degeneration. Also, ASH reverses acetylcholinesterase activity and thus has potential to modulate cholinergic function or may be connected to clearing effect associated with the degradation of Aβ by many proteases, including neprilysin, endothelin-converting enzyme, angiotensin-converting enzyme, plasminogen activator and matrix metalloproteinase-9 [56]–[60]. ASH extract used in the present study is known to contain several compounds besides withanolide A. However, it is reasonable to expect that appropriate combinations of multiple chemopreventive components might provide greater efficacy than the administration of individual component. It is unlikely that chemoprevention of AD can be achieved by a single agent. Accordingly, there is need to develop a mixed cocktail approach with multiple herbal ingredients that act in a concerted way and produce multiple cellular effects with further enhancement of the efficacy in a positive manner for the effective management of AD. Further studies of ASH will probably contribute to resolving an urgent unmet medical need involving efficacious treatments that may offer a cure for neurodegenerative diseases. Studies are in progress from this point of view.

## Conclusions

In summary, this study demonstrated that Methanol: Chloroform (3:1) extract prepared from the dried roots of ASH and comprising alkaloids and steroidal lactones, the prominent being Withanolide A, was neuroprotective against Aβ-induced cytotoxicity. This observation was supported by trypan blue staining of the dead cells, cellular localization, MTT formazan exocytosis, levels of acetylcholinesterase activity and % cell viability confirming the chemopreventive or protective effects of ASH against Aβ induced toxicity (Fig.9). Further, the reduction in the levels of MAP2 in ASH treated cells and decreasing its levels in HIV-1 infected and drugs of abuse treated cells suggests that ASH could be a potential novel drug to reduce the brain amyloid burden and/or improve the HIV-1 associated neurocognitive impairments (Fig.9). The studies carried out with ASH purified constituent WA showed similar neuroprotective properties in neuronal cells. These results further established that neuroprotective properties of the ASH root extract observed in the present study may provide some explanation for the ethnopharmacological uses of ASH in traditional medicine for cognitive and other HIV associated neurodegenerative disorders.
